# Tris(4-methyl­anilinium) penta­chlorido­anti­monate(III) chloride monohydrate

**DOI:** 10.1107/S1600536812009427

**Published:** 2012-03-17

**Authors:** Qian Xu

**Affiliations:** a Ordered Matter Science Research Center, College of Chemistry and Chemical Engineering, Southeast University, Nanjing 211189, People’s Republic of China

## Abstract

The title compound, (C_7_H_10_N)_3_[SbCl_5_]Cl·H_2_O, consists of 4-methyl­anilinium cations, Cl^−^ and [SbCl_5_]^2−^ anions and water mol­ecules. The five Cl atoms bound to Sb [Sb—Cl = 2.4043 (9)–2.6262 (11) Å] form a square-pyramidal coordination environment. In addition, two [SbCl_5_]^2−^ anions related by an inversion center are joined by Sb⋯Cl inter­actions [Sb⋯Cl = 3.7273 (14) Å] into an [Sb_2_Cl_10_]^4−^ dimer with two bridging Cl atoms. The anions, water mol­ecules and ammonium groups of the cations are linked by N—H⋯Cl, N—H⋯O and O—H⋯Cl hydrogen bonds, forming layers parallel to the *ac* plane. The benzene rings of the 4-methyl­anilinium cations are packed between these layers.

## Related literature

For the closely related structures of bis­(anilinium) penta­chlorido­anti­monate(III) and tris­(anilinium) chloride penta­chlorido­anti­monate(III) monohydrate, see: Lipka (1980 ▶) and Chaabouni *et al.* (2004 ▶), respectively.
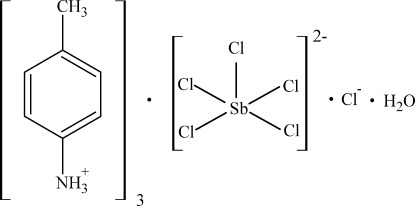


## Experimental

### 

#### Crystal data

(C_7_H_10_N)_3_[SbCl_5_]Cl·H_2_O
*M* *_r_* = 676.95
Triclinic, 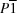

*a* = 9.4109 (19) Å
*b* = 12.867 (3) Å
*c* = 13.501 (3) Å
α = 63.35 (3)°
β = 83.08 (3)°
γ = 82.51 (3)°
*V* = 1445.1 (5) Å^3^
*Z* = 2
Mo *K*α radiation
μ = 1.53 mm^−1^
*T* = 293 K
0.31 × 0.25 × 0.22 mm

#### Data collection

Rigaku SCXmini diffractometer
Absorption correction: multi-scan (*CrystalClear*; Rigaku, 2005 ▶) *T*_min_ = 0.649, *T*_max_ = 0.730
15103 measured reflections
6609 independent reflections
6031 reflections with *I* > 2σ(*I*)
*R*_int_ = 0.029

#### Refinement

*R*[*F* ^2^ > 2σ(*F* ^2^)] = 0.028
*wR*(*F* ^2^) = 0.075
*S* = 1.06
6609 reflections
304 parameters
3 restraints
H atoms treated by a mixture of independent and constrained refinement
Δρ_max_ = 0.45 e Å^−3^
Δρ_min_ = −0.63 e Å^−3^

### 

Data collection: *CrystalClear* (Rigaku, 2005 ▶); cell refinement: *CrystalClear*; data reduction: *CrystalClear*; program(s) used to solve structure: *SHELXS97* (Sheldrick, 2008 ▶); program(s) used to refine structure: *SHELXL97* (Sheldrick, 2008 ▶); molecular graphics: *DIAMOND* (Brandenburg & Putz, 2005 ▶); software used to prepare material for publication: *SHELXL97*.

## Supplementary Material

Crystal structure: contains datablock(s) I, global. DOI: 10.1107/S1600536812009427/yk2046sup1.cif

Structure factors: contains datablock(s) I. DOI: 10.1107/S1600536812009427/yk2046Isup2.hkl

Additional supplementary materials:  crystallographic information; 3D view; checkCIF report

## Figures and Tables

**Table 1:** Hydrogen-bond geometry (Å, °)

*D*—H⋯*A*	*D*—H	H⋯*A*	*D*⋯*A*	*D*—H⋯*A*
N1—H1*D*⋯Cl1	0.89	2.32	3.202 (2)	170
N1—H1*E*⋯Cl1^i^	0.89	2.43	3.298 (3)	167
N1—H1*F*⋯Cl3^i^	0.89	2.52	3.381 (2)	162
N2—H2*A*⋯O1*W*	0.89	1.97	2.849 (4)	170
N2—H2*B*⋯Cl3^ii^	0.89	2.61	3.389 (2)	146
N2—H2*C*⋯Cl4	0.89	2.45	3.307 (3)	161
N3—H3*A*⋯Cl1^i^	0.89	2.46	3.293 (3)	156
N3—H3*B*⋯Cl5	0.89	2.51	3.402 (2)	174
N3—H3*C*⋯Cl1^iii^	0.89	2.32	3.201 (3)	173
O1*W*—H1*WA*⋯Cl4^ii^	0.85 (1)	2.56 (3)	3.283 (2)	143 (4)
O1*W*—H1*WB*⋯Cl2^iv^	0.85 (4)	2.69 (3)	3.344 (2)	136 (4)

Symmetry codes: (i) 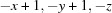; (ii) 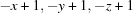; (iii) 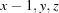; (iv) 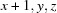.

**Fig. 1.**
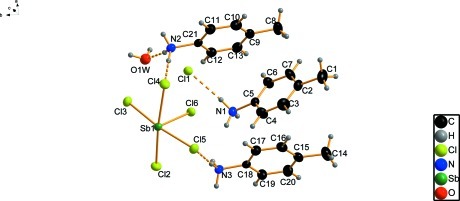
The asymmetric unit of the title compound with atom labelling scheme and thermal ellipsoids drawn at the 30% probability level. Hydrogen bonds are shown as dashed lines.

**Fig. 2.**
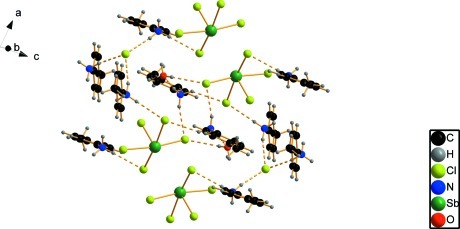
The system of hydrogen bonds (shown as dashed lines) in the title compound.

## supplementary crystallographic information

### Comment

The title compound was prepared in attempts to synthesize new materials with ferroelectric phase transition. Unfortunately, the dielectric permeability of title compound goes smoothly in the temperature range 80–293 K, suggesting no distinct phase transitions occurred.

A view asymmetric unit of the title compound is shown in Fig. 1. It consists of three p-toluidine cations, one water molecule, and Cl^-^ and [SbCl_5_]^2-^ anions. Hydrogen bonding between cations, anions and water molecules (Table 1, Fig. 2) produces two-dimensional network parallel to the *ac* plane. Analogous system of hydrogen bonds exists in closely related structure of tris-anilinium chloride pentachloroantimonate (III) monohydrate (Chaabouni *et al.*, 2004)

The Sb atom is coordinated by five Cl atoms in a slightly distorted square-pyramidal mode. The distance from Sb to the apical Cl atom is much shorter than the equatorial Sb—Cl bonds. Such structure is typical of [SbCl_5_]^2-^-containing salts (Lipka, 1980). Two anions [SbCl_5_]^2-^ related by an inversion center are joined by Sb···Cl interactions [Sb···Cl = 3.7273 (14) Å] into the Sb_2_Cl_10_^4-^ dimer with two bridging Cl atoms.

### Experimental

The mixture of SbCl_3_ (1.1 g, 5 mmol) and p-toluidine (1.05 g, 10 mmol) was dissolved in hydrochloric acid and stirred for several minutes at room temperature. Colorless crystals suitable for X-ray diffraction analysis were obtained by slow evaporation of the solution at room temperature over 2 weeks.

### Refinement

All H atoms were placed in geometrically idealized positions and constrained to ride on their parent atoms with C—H distances of 0.93 Å for phenyl and 0.96 Å for methyl groups, and N—H = 0.89 Å; *U*_iso_(H) = 1.2 *U*_iso_(C) for phenyl and 1.5 U*_iso_*(C,N) for methyl and ammonium H atoms. In water molecule the O—H distances in water were restrained to 0.85 (1) Å, and the distance H···H – to 1.38 (2) Å.

### Crystal data

**Table:**

(C_7_H_10_N)_3_[SbCl_5_]Cl·H_2_O	*Z* = 2
*M**_r_* = 676.95	*F*(000) = 680
Triclinic, *P*1	*D*_x_ = 1.556 Mg m^−^^3^
Hall symbol: -P 1	Mo *K*α radiation, λ = 0.71073 Å
*a* = 9.4109 (19) Å	Cell parameters from 6609 reflections
*b* = 12.867 (3) Å	θ = 2.3–27.5°
*c* = 13.501 (3) Å	µ = 1.53 mm^−^^1^
α = 63.35 (3)°	*T* = 293 K
β = 83.08 (3)°	Block, brown
γ = 82.51 (3)°	0.31 × 0.25 × 0.22 mm
*V* = 1445.1 (5) Å^3^	

### Data collection

**Table:**

Rigaku SCXmini diffractometer	6609 independent reflections
Radiation source: fine-focus sealed tube	6031 reflections with *I* > 2σ(*I*)
Graphite monochromator	*R*_int_ = 0.029
ω scans	θ_max_ = 27.5°, θ_min_ = 3.1°
Absorption correction: multi-scan (*CrystalClear*; Rigaku, 2005)	*h* = −12→12
*T*_min_ = 0.649, *T*_max_ = 0.730	*k* = −16→16
15103 measured reflections	*l* = −17→17

### Refinement

**Table:**

Refinement on *F*^2^	Secondary atom site location: difference Fourier map
Least-squares matrix: full	Hydrogen site location: inferred from neighbouring sites
*R*[*F*^2^ > 2σ(*F*^2^)] = 0.028	H atoms treated by a mixture of independent and constrained refinement
*wR*(*F*^2^) = 0.075	*w* = 1/[σ^2^(*F*_o_^2^) + (0.0383*P*)^2^ + 0.2569*P*] where *P* = (*F*_o_^2^ + 2*F*_c_^2^)/3
*S* = 1.06	(Δ/σ)_max_ = 0.041
6609 reflections	Δρ_max_ = 0.45 e Å^−^^3^
304 parameters	Δρ_min_ = −0.63 e Å^−^^3^
3 restraints	Extinction correction: *SHELXL97* (Sheldrick, 2008), Fc^*^=kFc[1+0.001xFc^2^λ^3^/sin(2θ)]^-1/4^
Primary atom site location: structure-invariant direct methods	Extinction coefficient: 0.0277 (8)

### Special details

**Table:**

Geometry. All e.s.d.'s (except the e.s.d. in the dihedral angle between two l.s. planes) are estimated using the full covariance matrix. The cell e.s.d.'s are taken into account individually in the estimation of e.s.d.'s in distances, angles and torsion angles; correlations between e.s.d.'s in cell parameters are only used when they are defined by crystal symmetry. An approximate (isotropic) treatment of cell e.s.d.'s is used for estimating e.s.d.'s involving l.s. planes.
Refinement. Refinement of *F*^2^ against ALL reflections. The weighted *R*-factor *wR* and goodness of fit *S* are based on *F*^2^, conventional *R*-factors *R* are based on *F*, with *F* set to zero for negative *F*^2^. The threshold expression of *F*^2^ > σ(*F*^2^) is used only for calculating *R*-factors(gt) *etc*. and is not relevant to the choice of reflections for refinement. *R*-factors based on *F*^2^ are statistically about twice as large as those based on *F*, and *R*- factors based on ALL data will be even larger.

### Fractional atomic coordinates and isotropic or equivalent isotropic displacement parameters (Å^2^)

**Table:**

	*x*	*y*	*z*	*U*_iso_*/*U*_eq_	
C1	0.5819 (5)	−0.1182 (3)	0.1786 (3)	0.0788 (11)	
H1A	0.6179	−0.1519	0.1289	0.118*	
H1B	0.4873	−0.1416	0.2087	0.118*	
H1C	0.6445	−0.1446	0.2379	0.118*	
C2	0.5756 (3)	0.0133 (2)	0.1160 (2)	0.0491 (6)	
C3	0.4543 (4)	0.0833 (3)	0.1201 (3)	0.0681 (9)	
H3	0.3746	0.0492	0.1655	0.082*	
C4	0.4473 (3)	0.2033 (3)	0.0584 (3)	0.0599 (8)	
H4	0.3635	0.2493	0.0612	0.072*	
C5	0.5651 (3)	0.2528 (2)	−0.00645 (19)	0.0379 (5)	
C6	0.6881 (3)	0.1871 (2)	−0.0109 (3)	0.0553 (7)	
H6	0.7685	0.2219	−0.0545	0.066*	
C7	0.6920 (4)	0.0668 (2)	0.0508 (3)	0.0614 (8)	
H7	0.7761	0.0214	0.0476	0.074*	
C8	0.7153 (4)	−0.0700 (3)	0.4215 (4)	0.0913 (13)	
H8A	0.8016	−0.1084	0.4582	0.137*	
H8B	0.7278	−0.0594	0.3460	0.137*	
H8C	0.6367	−0.1169	0.4594	0.137*	
C9	0.6836 (3)	0.0481 (3)	0.4229 (3)	0.0570 (8)	
C10	0.6689 (3)	0.0565 (3)	0.5211 (3)	0.0636 (8)	
H10	0.6762	−0.0115	0.5872	0.076*	
C11	0.6434 (3)	0.1633 (2)	0.5252 (2)	0.0522 (7)	
H11	0.6358	0.1673	0.5927	0.063*	
C12	0.6420 (3)	0.2577 (2)	0.3272 (2)	0.0486 (6)	
H12	0.6318	0.3255	0.2613	0.058*	
C13	0.6697 (3)	0.1498 (3)	0.3258 (3)	0.0595 (8)	
H13	0.6791	0.1459	0.2582	0.071*	
C14	0.0597 (5)	−0.1055 (3)	0.2261 (4)	0.0835 (11)	
H14A	−0.0081	−0.1487	0.2850	0.125*	
H14B	0.1549	−0.1419	0.2437	0.125*	
H14C	0.0376	−0.1044	0.1580	0.125*	
C15	0.0512 (3)	0.0175 (2)	0.2129 (3)	0.0520 (7)	
C16	0.0510 (3)	0.0397 (2)	0.3037 (2)	0.0548 (7)	
H16	0.0582	−0.0228	0.3738	0.066*	
C17	0.0403 (3)	0.1524 (2)	0.2937 (2)	0.0504 (6)	
H17	0.0406	0.1655	0.3561	0.060*	
C18	0.0292 (2)	0.2445 (2)	0.1900 (2)	0.0379 (5)	
C19	0.0306 (3)	0.2262 (2)	0.0970 (2)	0.0509 (7)	
H19	0.0239	0.2889	0.0271	0.061*	
C20	0.0423 (4)	0.1126 (3)	0.1097 (3)	0.0597 (8)	
H20	0.0443	0.0997	0.0470	0.072*	
C21	0.6298 (2)	0.2630 (2)	0.4271 (2)	0.0382 (5)	
Cl6	0.28747 (6)	0.39064 (5)	0.30055 (5)	0.03994 (14)	
Cl3	0.34735 (7)	0.63633 (5)	0.32019 (5)	0.04439 (14)	
Cl2	0.02743 (7)	0.61394 (6)	0.19317 (5)	0.04800 (15)	
Cl4	0.27679 (8)	0.36960 (6)	0.56178 (5)	0.05163 (16)	
Cl5	−0.04581 (7)	0.34297 (6)	0.43914 (6)	0.04692 (15)	
N1	0.5587 (2)	0.38021 (17)	−0.07764 (17)	0.0446 (5)	
H1D	0.6201	0.4117	−0.0559	0.067*	
H1E	0.4701	0.4118	−0.0718	0.067*	
H1F	0.5820	0.3938	−0.1480	0.067*	
N2	0.5985 (2)	0.37651 (19)	0.43079 (19)	0.0467 (5)	
H2A	0.6203	0.4335	0.3642	0.070*	
H2B	0.6507	0.3784	0.4806	0.070*	
H2C	0.5056	0.3862	0.4498	0.070*	
N3	0.0109 (2)	0.36535 (18)	0.17691 (19)	0.0454 (5)	
H3A	0.0890	0.4014	0.1407	0.068*	
H3B	−0.0019	0.3647	0.2436	0.068*	
H3C	−0.0653	0.4029	0.1385	0.068*	
Sb1	0.137115 (15)	0.499310 (12)	0.384947 (11)	0.03139 (7)	
Cl1	0.74902 (6)	0.49450 (5)	0.02248 (5)	0.04278 (14)	
O1W	0.6775 (2)	0.5732 (2)	0.23099 (19)	0.0591 (5)	
H1WA	0.686 (5)	0.620 (4)	0.258 (4)	0.129 (18)*	
H1WB	0.758 (3)	0.562 (4)	0.200 (4)	0.14 (2)*	

### Atomic displacement parameters (Å^2^)

**Table:**

	*U*^11^	*U*^22^	*U*^33^	*U*^12^	*U*^13^	*U*^23^
C1	0.126 (3)	0.0427 (16)	0.059 (2)	−0.0255 (19)	−0.015 (2)	−0.0082 (14)
C2	0.0684 (18)	0.0365 (13)	0.0381 (14)	−0.0169 (13)	−0.0052 (12)	−0.0091 (11)
C3	0.0575 (19)	0.0624 (19)	0.064 (2)	−0.0254 (16)	0.0151 (15)	−0.0092 (15)
C4	0.0463 (15)	0.0519 (17)	0.0643 (19)	−0.0039 (13)	0.0102 (13)	−0.0138 (14)
C5	0.0428 (13)	0.0362 (12)	0.0348 (12)	−0.0074 (10)	−0.0032 (10)	−0.0146 (10)
C6	0.0432 (14)	0.0420 (14)	0.0656 (19)	−0.0089 (12)	0.0110 (13)	−0.0125 (13)
C7	0.0587 (18)	0.0377 (14)	0.073 (2)	0.0026 (13)	0.0010 (15)	−0.0146 (14)
C8	0.063 (2)	0.061 (2)	0.167 (4)	−0.0060 (18)	−0.007 (2)	−0.066 (3)
C9	0.0355 (13)	0.0500 (16)	0.092 (2)	−0.0043 (12)	−0.0065 (14)	−0.0361 (16)
C10	0.0588 (18)	0.0506 (17)	0.068 (2)	−0.0120 (14)	−0.0118 (15)	−0.0110 (15)
C11	0.0547 (16)	0.0536 (16)	0.0476 (16)	−0.0097 (13)	−0.0041 (12)	−0.0204 (13)
C12	0.0460 (14)	0.0525 (16)	0.0477 (15)	−0.0012 (12)	−0.0047 (11)	−0.0228 (12)
C13	0.0535 (17)	0.072 (2)	0.070 (2)	−0.0007 (15)	−0.0069 (14)	−0.0470 (17)
C14	0.094 (3)	0.054 (2)	0.102 (3)	0.0050 (19)	−0.003 (2)	−0.038 (2)
C15	0.0428 (14)	0.0463 (15)	0.0651 (18)	0.0038 (12)	−0.0050 (13)	−0.0245 (13)
C16	0.0547 (16)	0.0446 (15)	0.0519 (17)	−0.0005 (13)	−0.0095 (13)	−0.0091 (12)
C17	0.0586 (16)	0.0506 (15)	0.0418 (14)	−0.0059 (13)	−0.0096 (12)	−0.0184 (12)
C18	0.0321 (11)	0.0394 (12)	0.0441 (13)	−0.0036 (10)	−0.0078 (10)	−0.0186 (10)
C19	0.0662 (18)	0.0444 (14)	0.0394 (14)	−0.0025 (13)	−0.0103 (12)	−0.0150 (11)
C20	0.076 (2)	0.0608 (18)	0.0523 (17)	0.0000 (16)	−0.0066 (15)	−0.0344 (15)
C21	0.0283 (11)	0.0415 (13)	0.0487 (14)	−0.0063 (10)	−0.0029 (10)	−0.0225 (11)
Cl6	0.0361 (3)	0.0465 (3)	0.0429 (3)	−0.0003 (2)	0.0002 (2)	−0.0262 (3)
Cl3	0.0448 (3)	0.0446 (3)	0.0474 (3)	−0.0162 (3)	0.0032 (3)	−0.0218 (3)
Cl2	0.0510 (4)	0.0476 (3)	0.0368 (3)	−0.0034 (3)	−0.0084 (3)	−0.0099 (3)
Cl4	0.0647 (4)	0.0520 (4)	0.0348 (3)	0.0012 (3)	−0.0120 (3)	−0.0156 (3)
Cl5	0.0387 (3)	0.0489 (3)	0.0551 (4)	−0.0166 (3)	0.0054 (3)	−0.0233 (3)
N1	0.0557 (13)	0.0341 (10)	0.0411 (12)	−0.0069 (9)	−0.0067 (9)	−0.0124 (9)
N2	0.0415 (11)	0.0518 (13)	0.0542 (13)	−0.0025 (10)	−0.0072 (10)	−0.0293 (11)
N3	0.0459 (12)	0.0422 (11)	0.0508 (13)	−0.0045 (10)	−0.0130 (10)	−0.0202 (10)
Sb1	0.02980 (10)	0.03616 (10)	0.02999 (10)	−0.00388 (6)	0.00011 (6)	−0.01637 (7)
Cl1	0.0404 (3)	0.0455 (3)	0.0445 (3)	−0.0007 (3)	−0.0049 (2)	−0.0220 (3)
O1W	0.0567 (13)	0.0671 (14)	0.0617 (13)	−0.0053 (11)	−0.0021 (10)	−0.0361 (11)

### Geometric parameters (Å, º)

**Table:**

C1—C2	1.511 (4)	C14—H14B	0.9600
C1—H1A	0.9600	C14—H14C	0.9600
C1—H1B	0.9600	C15—C16	1.377 (4)
C1—H1C	0.9600	C15—C20	1.385 (4)
C2—C7	1.367 (4)	C16—C17	1.386 (4)
C2—C3	1.371 (5)	C16—H16	0.9300
C3—C4	1.385 (4)	C17—C18	1.375 (4)
C3—H3	0.9300	C17—H17	0.9300
C4—C5	1.360 (4)	C18—C19	1.376 (3)
C4—H4	0.9300	C18—N3	1.473 (3)
C5—C6	1.353 (4)	C19—C20	1.385 (4)
C5—N1	1.480 (3)	C19—H19	0.9300
C6—C7	1.388 (4)	C20—H20	0.9300
C6—H6	0.9300	C21—N2	1.474 (3)
C7—H7	0.9300	Cl6—Sb1	2.4043 (9)
C8—C9	1.518 (4)	Cl3—Sb1	2.6262 (11)
C8—H8A	0.9600	Cl2—Sb1	2.6011 (12)
C8—H8B	0.9600	Cl4—Sb1	2.6120 (13)
C8—H8C	0.9600	Cl5—Sb1	2.6124 (11)
C9—C10	1.367 (5)	N1—H1D	0.8900
C9—C13	1.380 (5)	N1—H1E	0.8900
C10—C11	1.389 (4)	N1—H1F	0.8900
C10—H10	0.9300	N2—H2A	0.8900
C11—C21	1.375 (4)	N2—H2B	0.8900
C11—H11	0.9300	N2—H2C	0.8900
C12—C21	1.371 (3)	N3—H3A	0.8900
C12—C13	1.387 (4)	N3—H3B	0.8900
C12—H12	0.9300	N3—H3C	0.8900
C13—H13	0.9300	O1W—H1WA	0.845 (10)
C14—C15	1.504 (4)	O1W—H1WB	0.847 (10)
C14—H14A	0.9600		
			
C2—C1—H1A	109.5	C16—C15—C20	117.5 (3)
C2—C1—H1B	109.5	C16—C15—C14	121.0 (3)
H1A—C1—H1B	109.5	C20—C15—C14	121.5 (3)
C2—C1—H1C	109.5	C15—C16—C17	121.9 (3)
H1A—C1—H1C	109.5	C15—C16—H16	119.0
H1B—C1—H1C	109.5	C17—C16—H16	119.0
C7—C2—C3	117.4 (2)	C18—C17—C16	118.8 (3)
C7—C2—C1	120.5 (3)	C18—C17—H17	120.6
C3—C2—C1	122.2 (3)	C16—C17—H17	120.6
C2—C3—C4	121.8 (3)	C17—C18—C19	121.1 (2)
C2—C3—H3	119.1	C17—C18—N3	120.3 (2)
C4—C3—H3	119.1	C19—C18—N3	118.6 (2)
C5—C4—C3	118.8 (3)	C18—C19—C20	118.7 (2)
C5—C4—H4	120.6	C18—C19—H19	120.7
C3—C4—H4	120.6	C20—C19—H19	120.7
C6—C5—C4	121.3 (2)	C15—C20—C19	121.9 (3)
C6—C5—N1	118.6 (2)	C15—C20—H20	119.0
C4—C5—N1	120.1 (2)	C19—C20—H20	119.0
C5—C6—C7	118.8 (3)	C12—C21—C11	121.0 (2)
C5—C6—H6	120.6	C12—C21—N2	120.1 (2)
C7—C6—H6	120.6	C11—C21—N2	118.9 (2)
C2—C7—C6	121.9 (3)	C5—N1—H1D	109.5
C2—C7—H7	119.0	C5—N1—H1E	109.5
C6—C7—H7	119.0	H1D—N1—H1E	109.5
C9—C8—H8A	109.5	C5—N1—H1F	109.5
C9—C8—H8B	109.5	H1D—N1—H1F	109.5
H8A—C8—H8B	109.5	H1E—N1—H1F	109.5
C9—C8—H8C	109.5	C21—N2—H2A	109.5
H8A—C8—H8C	109.5	C21—N2—H2B	109.5
H8B—C8—H8C	109.5	H2A—N2—H2B	109.5
C10—C9—C13	118.1 (3)	C21—N2—H2C	109.5
C10—C9—C8	120.5 (3)	H2A—N2—H2C	109.5
C13—C9—C8	121.4 (3)	H2B—N2—H2C	109.5
C9—C10—C11	122.0 (3)	C18—N3—H3A	109.5
C9—C10—H10	119.0	C18—N3—H3B	109.5
C11—C10—H10	119.0	H3A—N3—H3B	109.5
C21—C11—C10	118.5 (3)	C18—N3—H3C	109.5
C21—C11—H11	120.8	H3A—N3—H3C	109.5
C10—C11—H11	120.8	H3B—N3—H3C	109.5
C21—C12—C13	119.1 (3)	Cl6—Sb1—Cl2	85.80 (4)
C21—C12—H12	120.5	Cl6—Sb1—Cl4	85.36 (3)
C13—C12—H12	120.5	Cl2—Sb1—Cl4	171.07 (2)
C9—C13—C12	121.3 (3)	Cl6—Sb1—Cl5	86.20 (3)
C9—C13—H13	119.4	Cl2—Sb1—Cl5	88.98 (4)
C12—C13—H13	119.4	Cl4—Sb1—Cl5	91.76 (4)
C15—C14—H14A	109.5	Cl6—Sb1—Cl3	85.26 (3)
C15—C14—H14B	109.5	Cl2—Sb1—Cl3	91.42 (4)
H14A—C14—H14B	109.5	Cl4—Sb1—Cl3	86.53 (4)
C15—C14—H14C	109.5	Cl5—Sb1—Cl3	171.40 (2)
H14A—C14—H14C	109.5	H1WA—O1W—H1WB	109 (2)
H14B—C14—H14C	109.5		
			
C7—C2—C3—C4	1.8 (5)	C21—C12—C13—C9	0.7 (4)
C1—C2—C3—C4	−176.9 (3)	C20—C15—C16—C17	0.9 (4)
C2—C3—C4—C5	−1.1 (5)	C14—C15—C16—C17	−178.7 (3)
C3—C4—C5—C6	−0.4 (5)	C15—C16—C17—C18	0.2 (4)
C3—C4—C5—N1	176.9 (3)	C16—C17—C18—C19	−1.0 (4)
C4—C5—C6—C7	1.1 (5)	C16—C17—C18—N3	177.2 (2)
N1—C5—C6—C7	−176.3 (3)	C17—C18—C19—C20	0.6 (4)
C3—C2—C7—C6	−1.1 (5)	N3—C18—C19—C20	−177.6 (3)
C1—C2—C7—C6	177.6 (3)	C16—C15—C20—C19	−1.3 (5)
C5—C6—C7—C2	−0.3 (5)	C14—C15—C20—C19	178.2 (3)
C13—C9—C10—C11	−1.2 (4)	C18—C19—C20—C15	0.6 (5)
C8—C9—C10—C11	178.4 (3)	C13—C12—C21—C11	−0.5 (4)
C9—C10—C11—C21	1.4 (4)	C13—C12—C21—N2	−179.2 (2)
C10—C9—C13—C12	0.2 (4)	C10—C11—C21—C12	−0.5 (4)
C8—C9—C13—C12	−179.4 (3)	C10—C11—C21—N2	178.2 (2)

### Hydrogen-bond geometry (Å, º)

**Table:**

*D*—H···*A*	*D*—H	H···*A*	*D*···*A*	*D*—H···*A*
N1—H1*D*···Cl1	0.89	2.32	3.202 (2)	170
N1—H1*E*···Cl1^i^	0.89	2.43	3.298 (3)	167
N1—H1*F*···Cl3^i^	0.89	2.52	3.381 (2)	162
N2—H2*A*···O1*W*	0.89	1.97	2.849 (4)	170
N2—H2*B*···Cl3^ii^	0.89	2.61	3.389 (2)	146
N2—H2*C*···Cl4	0.89	2.45	3.307 (3)	161
N3—H3*A*···Cl1^i^	0.89	2.46	3.293 (3)	156
N3—H3*B*···Cl5	0.89	2.51	3.402 (2)	174
N3—H3*C*···Cl1^iii^	0.89	2.32	3.201 (3)	173
O1*W*—H1*WA*···Cl4^ii^	0.85 (1)	2.56 (3)	3.283 (2)	143 (4)
O1*W*—H1*WB*···Cl2^iv^	0.85 (4)	2.69 (3)	3.344 (2)	136 (4)

Symmetry codes: (i) −*x*+1, −*y*+1, −*z*; (ii) −*x*+1, −*y*+1, −*z*+1; (iii) *x*−1, *y*, *z*; (iv) *x*+1, *y*, *z*.
